# New insights into the pathogenicity of non-synonymous variants through multi-level analysis

**DOI:** 10.1038/s41598-018-38189-9

**Published:** 2019-02-07

**Authors:** Hong Sun, Guangjun Yu

**Affiliations:** Shanghai Children’s Hospital, Shanghai Jiao Tong University, Shanghai, 200062 China

## Abstract

Precise classification of non-synonymous single nucleotide variants (SNVs) is a fundamental goal of clinical genetics. Next-generation sequencing technology is effective for establishing the basis of genetic diseases. However, identification of variants that are causal for genetic diseases remains a challenge. We analyzed human non-synonymous SNVs from a multilevel perspective to characterize pathogenicity. We showed that computational tools, though each having its own strength and weakness, tend to be overly dependent on the degree of conservation. For the mutations at non-degenerate sites, the amino acid sites of pathogenic substitutions show a distinct distribution in the classes of protein domains compared with the sites of benign substitutions. Overlooked disease susceptibility of genes explains in part the failures of computational tools. The more pathogenic sites observed, the more likely the gene is expressed in a high abundance or in a high tissue-specific manner, and have a high node degree of protein-protein interaction. The destroyed functions due to some false-negative mutations may arise because of a reprieve from the epigenetic repressed state which shouldn’t happen in multiple biological conditions, instead of the defective protein. Our work adds more to our knowledge of non-synonymous SNVs’ pathogenicity, thus will benefit the field of clinical genetics.

## Introduction

Single nucleotide variants (SNVs) are among the most frequent and widespread changes in the genome^1^. Most of these changes are functionally neutral, however, some variants produce dramatic phenotype and may lead to various diseases as a consequence^2^. Approximately half of the known inherited disease mutations stems from non-synonymous SNVs^3^, which may destroy the function of the encoded proteins, thereby causing diseases. Precise identification of non-synonymous SNVs causing human diseases will provide crucial insights directly affecting the clinical diagnosis and management of affected individuals.

Next-generation sequencing technology is a powerful and efficient means to comprehensively delineate the map of genetic variations^4^. In particular, exome sequencing has been demonstrated as an effective way to detect pathogenic non-synonymous SNVs underlying both Mendelian diseases^3^ as well as complex traits^5^. Clinical sequencing has been put into medical practice^6^, and it has been proven to be an effective alternative for identifying the genetic basis of diseases^7,8^. However, elucidating the associations between mutations and disease, though vastly important, is restricted by the difficulty in distinguishing pathogenic mutations from those that are functionally neutral. Therefore, computational prediction tools became preferred for prioritizing causal mutations.

Multiple computational methods have been developed for predicting pathogenicity, such as SIFT^9^, PROVEAN^10^, MutationTaster^11^, FATHMM-MKL^12^, FATHMM-XF^13^, FATHMM^14^, MetaSVM^15^, MetaLR^15^, PolyPhen-2^16^, MutationAssessor^17^, CADD^18^ and DANN^19^ *etc*. While these tools are commonly used to predict pathogenicity, these programs vary widely in their original purposes and the methods utilized. Some tools measure sequence conservation (e.g., SIFT), some assess the impact of variants on protein structure or function (e.g., PolyPhen-2), some try to quantify the overall pathogenic potential of a variant based on diverse types of genomic information (e.g., CADD) *etc*. Some tools integrate multiple scoring values for classification utilizing support vector machine (e.g., MetaSVM) or logistic regression (e.g., MetaLR), some other methods classify variants according to Bayesian methods (e.g., PolyPhen2), or mathematical operations (SIFT) *etc*. The dbNSFP algorithm integrates the output of different prediction tools so as to yield a single consensus prediction to facilitate comparison between scores^20^.

Despite the constant emergence of new computational methods to catalog human genetic variations, identification of variants that are causal for diseases remains a difficult task. Predictions made by different computational tools differ greatly when applied to the same variant^21^. It has been reported that around 73% of functional predictions are not effectively differentiated from neutral mutations, suggesting the high rate of false positive predictions^22^. Low specificity of characteristics will inevitably return many false predictions^22^. High accuracy is insufficient to indicate a good classifier as both false-positive and false-negative results can lead to serious consequences^23^. It is vital to better understand accuracies and limitations of the computational methods because published performance is confounded by serious problems, especially for some variants which are unlikely to cause monogenic diseases but are relevant to diseases in a more complex basis. In this work, we present some new insights to the pathogenicity of non-synonymous SNVs that would benefit the research community.

## Results

Identifying the genetic variants responsible for diseases is a major challenge of clinical whole exome sequencing. Multiple algorithms have been developed to distinguish pathogenic mutations from a large number of background variations based on different information of the variants. However, predictions made by different computational tools differ greatly when applied to the same variant^21^ and their relative merits and limitations are still unclear in practical applications.

To identify possible limitations in computational methods, we considered twelve computational tools because of their demonstrated fine performances, namely SIFT^9^, PROVEAN^10^, MutationTaster^11^, FATHMM-MKL^12^, FATHMM-XF^13^, FATHMM^14^, MetaSVM^15^, MetaLR^15^, PolyPhen-2^16^, MutationAssessor^17^, CADD^18^, and DANN^19^. To facilitate comparison between scores, we downloaded rank scores from dbNSFP v3.0^20^, which is an integrated database of functional predictions from multiple algorithms.

Based on the ClinVar^24^ annotation terms, we classified the genomic sites into four groups: pathogenic sites if pathogenic evidence was presented to the change(s) at the site but no evidence of benign effect from an authoritative source, and for the benign sites vice versa; a site is called as ‘both’ (pathogenic or benign) if both pathogenic and benign variants were found, and a site is called as ‘other’ if neither pathogenic nor benign variants were found. Based on the HGMD^25^ annotation terms, we called pathologic variants if there is cogent evidence to support their disease causing effect as DM variants (*i.e*., Disease causing mutation).

### Predictions should be evaluated thoroughly

We first evaluate the agreement between computational tools. The performance of individual computational tools differ in the quality of predictions based on the ClinVar annotations (Figure S1 and Table S1), and this observation is consistent with previously reported results^26^. The proportion of non-synonymous SNVs that have consensus predicted results between algorithms varied from 35% to 96%. The data indicate that the differences in feature sets and algorithms used by the different computational tools are major factors that lead to inconsistent predictions (Figure S2).

It is intuitively appealing that combination of prediction tools may enhance the predictive accuracy. We, therefore, calculated the performance of combinations of computational tools to determine whether the accuracy was improved. The criterion to categorize a variant as pathogenic or non-pathogenic was that all the algorithms combined agree on the prediction. We next evaluated the performance of combined predictions corresponding to the number of algorithms combined. The predictive accuracy increased and reached optimal performance when two or three tools were combined, and then decrease as sensitivity decrease fast when more tools were added (Figure S3).

There are meta-predictors, such as MetaLR and MetaSVM^15^, which integrate multiple results from different tools using machine learning approaches, *i.e*. support vector machine and logistic regression. Though MetaSVM and MetaLR have high accuracy compared to the other tools analyzed in this study, their sensitivity is lower (Figure S1). We also found that MetaSVM and MetaLR show poor agreement with other predictors, despite that the two predictors themselves share the highest predictive consistency (Figure S2). Combination of several predictors using machine learning approach also has problems^27^.

The intuitive explanation for the poor degree of agreement is that computational tools make predictive errors, or they do a good job in predicting pathogenesis, for different variants. As each method has its own strength and weakness, we assume that for each method their competition superiority is associated with the specific features of the studied variants or genes. We, therefore, evaluate merits and limitations in computational predictions and analyze in a broader view the evidence that may implicate possible roles for the variants in pathogenesis.

### The evidence from conservation analysis needs to be treated with caution

Most computational methods review the degree of conservation at the affected genomic loci to estimate deleteriousness. Comparative sequence analysis is a powerful source of information regarding deleteriousness, however, ancestral sequences that have evolved slowly by chance are indistinguishable and that functional divergence will lessen the correlation between past constraint and present-day deleteriousness^28,29^.

To evaluate possible impacts of evolutionary constraint on the computational predictions, we first used the phastCons score as a measurement of evolutionary conservation and investigated the correlation between deleteriousness and phastCons scores derived with the parameters for the three species set (vertebrates, placental mammals, and primates). We found that some benign variants are located at positions highly conserved across vertebrate but less conserved among mammals or primates, and some are located at positions conserved among primates but not conserved when compared to non-primate vertebrates (Figure S4a,b). The same observation was found for pathogenic variants and DM variants (Figure S4e,f,i,j).

We further used the phyloP score as a measure of evolutional constraint. The phyloP scores represent as the log (P-value) under a null hypothesis of neutral evolution and can indicate both accelerated evolution as well as evolutionary conservation. As shown in Supplementary Figure S4, some benign variants are located at positions with positive phyloP scores, indicating conservation, and some are located at positions with negative phyloP scores, indicating fast-evolving. By contrast, most pathogenic variants are located at conserved positions indicated by the high positive phyloP scores (Figure S4g,h,k,l). It’s worth noting that some positions of pathogenic variants and DM variants are conserved when taking the vertebrate evolutionary branch into consideration, while appearing to be faster-evolving after primate speciation (Figure S4h,l).

**Figure 1:**
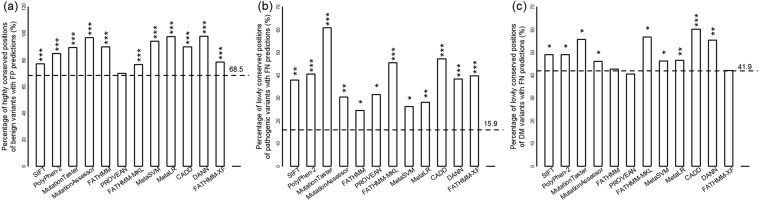
Overreliance on the degree of conservation in pathogenicity predictions. (**a**) Percentage of highly conserved positions of false-positive (FP) variants. (**b,c**) Percentage of lowly conserved positions of variants with false negative (FN) predictions for pathogenic variants annotated by ClinVar (**b**) and for DM variants annotated by HGMD (**c**). The dashed lines show the proportion of positions with high (**a**) or low (**b,c**) conservation score over all positions of benign (**a**) or pathogenic/DM (**b,c**) variants. The vertebrate phastCons score cutoff for high or low level conservation is set at 0.5. The observed excess for the positions are evaluated by p-values based on Pearson’s chi-squared test with respect to the proportion of all annotated positions of benign/pathogenic/DM variants. The significances are indicated as * for p < 0.05, ** for p < 10^−5^ and *** for p < 10^−10^.

For all the twelve computational models analyzed here, high proportion of benign variants at highly conserved positions are falsely predicted (Fig. 1a). Meanwhile, pathogenic/DM variants at the less conserved positions are more frequently predicted to be benign (Fig. 1b,c). The data indicates that computational tools are generally overly dependent on the conservation feature of the variants.

We further integrated the 1000 Genomes Project data^4^ to check possible pathogenesis of the mutations that change back to the ancestral state. Ancestral alleles of the single nucleotide polymorphism sites were inferred from a six-way primate alignment^4^. We found thirty-five mutations, which change back to the ancestral state, were assigned to be pathogenic either by ClinVar database or by HGMD database. These thirty-five mutations are located in thirty-three genes, and most of these human genes are highly conserved across species, *i.e*. only one copy of orthologues was found in many species based on the Ensembl gene annotation system^30^ (Table 1). GSEA analysis^31^ shows enrichment of these gene sets in GO_SYSTEM_PROCESS (Figure S5), which is a multicellular organismal process carried out by any of the organs or tissues in an organ system. The data suggest that the new mutant alleles are favored while the old alleles turn out to be detrimental, therefore it is possible that the corresponding process in human might have already adapted to changing to different environments. Though most mutations that change back to the ancestral state have a benign effect (Figure S6a) and they are enriched in the categories other than the disease causing category (Figure S6b), the fact that some of them will cause disorders calls us the attention that a good knowledge of the evolutionary roles of the associated genes will help in figuring out the causal alleles.

**Table 1:** Pathogenic mutations from derived allele to ancestral allele.

Symbol	No. of species		Sequence variant	ClinVar annotation	HGMD annotation*
	1:1 orthologue	1:many orthologue			
ABCA4	112	15	NM_000350.2:c.1268 A>G	Benign;Likely benign	DM
ABCC6	88	3	NM_001171.5:c.3961 G>A	Pathogenic	—
			NM_001171.5:c.1233 T>C	Likely benign	DM
ANK1	81	45	NM_000037.3:c.-108T>C	Pathogenic	DM?
ARSA	117	11	NM_000487.5:c.1178 C>G	Benign;Pathogenic	DP
ASPM	123	5	NM_018136.4:c.7787 T>C	Benign	DM
BCL11A	127	2	NM_018014.3:c.386-24278 G>A	Likely pathogenic	—
CBS	0	88	NM_000071.2:c.992 C>T	Pathogenic	—
CLCN7	130	1	NM_001287.5:c.1252 G>A	Benign	DM
COL4A4	86	12	NM_000092.4:c.3979 G>A	Likely pathogenic	—
CRYAB	118	8	NM_001885.2:c.166 C>T	Pathogenic	—
DHCR7	125	4	NM_001360.2:c.438 T>C	Benign	DM
DPYD	126	6	NM_000110.3:c.85 T>C	Pathogenic	DFP
DRAM2	116	13	NM_178454.4:c.131 G>A	Pathogenic	—
EYA1	130	2	NM_000503.5:c.1755T>C	Benign	DM
FBN1	113	12	NM_000138.4:c.2180 G>A	Pathogenic	—
FGFR1	88	43	NM_023110.2:c.899 T>C	Pathogenic	—
GJB2	69	14	NM_004004.5:c.487 A>G	Likely benign;Pathogenic	—
HEPACAM	81	48	NM_152722.4:c.274 C>T	Pathogenic	—
KEL	68	2	NM_000420.2:c.1790T>C	Pathogenic	FP
KRT14	61	0	NM_000526.4:c.369 T>C	—	DM
MAK	110	21	NM_001242957.1:c.37 G>A	Pathogenic	—
MYH7	109	9	NM_000257.3:c.5507 C>G	Pathogenic	—
NPHS1	121	3	NM_004646.3:c.1219 C>T	Likely pathogenic	—
OTOF	127	3	NM_194248.2:c.2736 G>C	Benign	DM
RAF1	112	16	NM_002880.3:c.781 C>G	Pathogenic	—
RARS2	132	0	NM_020320.3:c.953 G>A	Likely pathogenic	—
SCN5A	68	2	NM_198056.2:c.1673A>G	Benign;Pathogenic	DFP
SLC17A5	81	2	NM_012434.4:c.983 G>A	Likely pathogenic	—
SLC45A2	131	2	NM_016180.4:c.1122 C>G	Association;Protective	DM
			NM_016180.4:c.987 A>G	Benign	DM
SPG11	125	7	NM_025137.3:c.7023 C>T	Benign	DM
STAT1	117	14	NM_007315.3:c.494 A>G	Pathogenic	—
TAS2R16	25	1	NM_016945.2:c.516 T>G	Pathogenic;risk factor	DFP
XDH	128	6	NM_000379.3:c.3276 + 12 A>G	Likely pathogenic	—

*Abbreviations for HGMD annotation items: DM: Disease causing mutation; DM?: Disease causing mutation?; DP: Disease-associated polymorphism; DFP: Disease-associated polymorphism with supporting functional evidence; FP: *In vitro*/laboratory or *in vivo* functional polymorphism.

### Variation between prediction scores of the four alleles at non-degenerate sites

**Figure 2:**
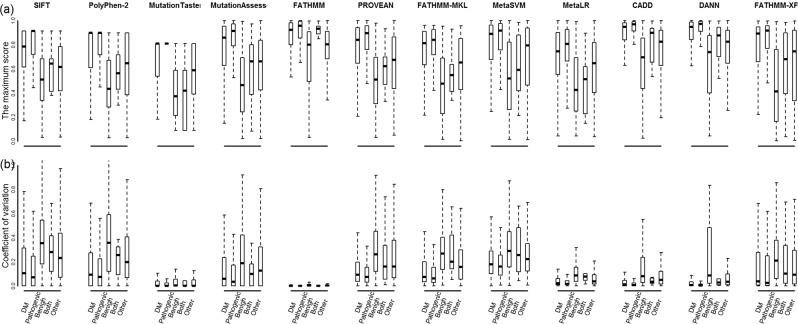
The maximum (**a**) and coefficient of variation (**b**) of prediction scores assigned to the four types of nucleotides at the non-degenerated sites corresponding to the four groups of variants annotated by ClinVar and DM variants annotated by HGMD. Wilcoxon tests were used to test the significance of the differences between groups of variants. Significant differences were observed between pathogenic variants and benign variants for all the computational tools.

For the non-degenerate sites, we first compared the maximum prediction score of the four different nucleotides to find the differences. We observed across all the twelve prediction tools that the maximum prediction score of pathogenic/DM sites is significantly higher than that of the other three groups of sites (Fig. 2a). We next examined the coefficient of variation (CV), which is a relative standard deviation to measure the degree of variation between the prediction scores of the four different nucleotides. The data showed that pathogenic/DM sites have much lower CVs compared to the other three groups of sites (Fig. 2b), indicating pathogenic/DM sites are more likely to be less tolerant to change itself rather than types of change in amino acid.

For the mutations at non-degenerate sites analyzed here, amino acid sites of pathogenic substitutions show a distinct distribution in the classes of protein domains compared to the sites of benign substitutions (Figure S7). As many computational methods use structural approaches to predict the functional effect of protein allelic variants, for the sites of pathogenic variants, the low CVs may indicate that any amino acid change would lead to change in the function of a protein domain.

We next investigated whether there exists any relationship between the degree of conservation and the predictions. For both sites of pathogenic/DM variants and sites of benign variants, Supplementary Figure S8 shows that there is no obvious correlation between conservation scores and the maximum prediction score, as well as between conservation scores and the CV score of the four prediction values. The data indicate that the conservation degree of a non-degenerate site may be less likely to influence the effects of mutations.

We further investigated the difference in conservation degree between the site groups using four conservation measurements, *i.e*. phastCons and phyloP scores derived from vertebrates or placental mammals model. The phastCons scores of sites of pathogenic variants are much higher and exhibit a remarkably narrow distribution compared to the sites of benign variants (Figure S9a); meanwhile, less conserved sites of pathogenic/DM variants (Figure S9f~m) and highly conserved sites of benign variants were also observed (Figure S9b~e). Both phastCons and phyloP scores can be used to measure conservation, the most important difference is that the phyloP scores reflect individual alignment columns. This property makes phyloP more appropriate than phastCons for evaluating signatures of selection at particular bases in the genome^32^. The difference in phyloP scores derived from placental mammals model is significant between sites of pathogenic and benign variants (Figure S9a), while no significant difference was observed in phyloP scores derived from vertebrates model (Figure S9a), indicating that some types of evolutionary events, occurring along branches of the mammalian phylogenetic tree, may bring certain information to the different clinical effects of mutations at some of these non-degenerate sites.

### Pathogenicity of variants is subject to disease susceptibility of the gene

Referring to the 1000 Genomes Project data^33^, we observed an obvious positive correlation between the occurrence frequency of nucleotide variations and the total length of the exons of a gene (Figure S10a), yet rates of SNV occurrence vary considerably among genes, from 15/Kb to 80/Kb with an average rate of 30/Kb. No obvious correlation was observed between the occurrence rate of nucleotide variations and the number of pathogenic/DM variations in genes (Figure S10b,c). Some genes contain as much or even more sites of pathogenic variants than the nucleotide variations detected by the 1000 Genomes Project, while in some other genes pathogenic variations are rarely observed relative to a large number of nucleotide variations. This difference between genes indicates that disease susceptibility of genes would be informative for determining the pathogenicity of a mutation occurred in that genes.

When considering all the variations in a gene as a whole, for the computational tools with relatively high false-positive rates, the false-negatively predicted variations gathered in certain specific genes, while the false-positively predicted variations increased with the increase of benign variations (Figure S11). A similar event was observed for the computational tools with relatively high false-negative rates. These observations indicated the necessity of gene-level analysis when making predictions.

It was suggested that it is important to know the sensitivity for variations in each gene/protein functional category^34,35^. We, therefore, investigated possible functional enrichment of genes having more or none pathogenic variations. We found that those genes, where no pathogenic variations were annotated neither by ClinVar database nor by HGMD database and more than fifty polymorphic sites were detected by the 1000 Genomes Project (disease-tolerant genes for short), are functionally enriched in transcriptional biomarkers of certain kinds of diseases etc. (GSEA analysis, FDR adjusted p-value < 0.05). Genes containing a high proportion (>30%) of pathogenic variations (disease-sensitive genes for short) are functionally enriched in cardiomyopathy, muscle filament sliding and muscle protein etc. (GSEA analysis, FDR adjusted p-value < 0.05). Overviews of GSEA analysis are illustrated in Supplementary Figure S12 for disease-sensitive genes and in Supplementary Figure S13 for disease-tolerant genes.

Computational tools encounter the same question in predictive accuracy when dealing with these two extreme types of genes, *i.e*. disease-tolerant genes and disease-sensitive genes (Figure S14**)**. We further used the online server, GAVIN^36^, which applies gene-specific thresholding for classifications to investigate the gene-specific disease susceptibility. Most of the disease-sensitive genes are assigned to GAVIN categories which is significantly predictive for pathogenicity. The data indicate that gene level analysis, *e.g*. protein functional analysis and analysis on disease susceptibility *etc*, is an important part of curating nucleotide variants to determine whether they are pathogenic.

When looking at the frequency of conserved nucleotide sites where variations were annotated by ClinVar database, we found that disease-sensitive genes contain more conserved sites than disease-tolerant genes, which is reasonable; however, they contain less conserved nucleotide sites when compared to all the other ClinVar genes out of these two extreme types of genes (Figure S15). This observation was yet another reminder that conservation criteria should be taken carefully when inferring possible pathogenesis of a mutation.

### Pathogenicity of nucleotide variations from the gene-level perspective

It was frequently suggested that candidate-gene prioritization could be developed based on gene-expression data or protein interactome based features^37,38^. Precise functions of genes are frequently dependent on the presence of their proteins expressed in a tissue-specific manner, and germ-line mutations causing the specific spatiotemporal damaged function of the genes are more likely to cause heritable diseases^39,40^.

**Figure 3:**
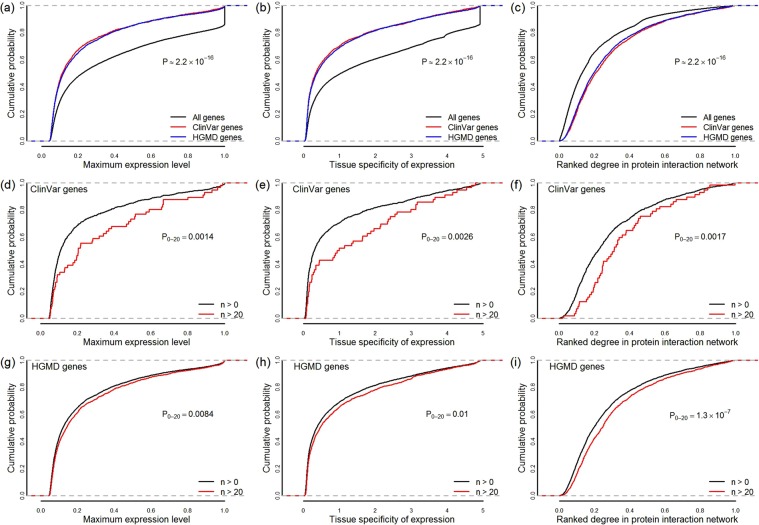
Characterization of pathogenicity at the gene level. Cumulative probability distributions of the maximum expression level (**a**) and the tissue specificity of expression (**b**) among the 53 human normal tissues examined, and the ranked protein-protein interaction network degree (**c**) for all genes, ClinVar genes and HGMD genes. (**d,e,f,g,h,i**) Analysis on ClinVar genes and HGMD genes corresponding to the number of pathogenic variants found in the gene. Cumulative probability distributions of the maximum expression level (**d,g**), the tissue specificity of expression (**e,h**) and the ranked protein-protein interaction network degree (**f,i**) for genes carrying at least one sites of pathogenic variants (n > 0) and genes carrying more than 20 sites of pathogenic variants. ClinVar genes are defined as genes that contain pathogenic variant(s) annotated by ClinVar database, and HGMD genes are defined as genes that contain DM variant(s) annotated by HGMD database. Kolmogorov-Smirnov tests were used to test the significance of the differences between gene groups. P-values from pairwise comparisons are shown.

We, therefore, examined the empirical cumulative distributions which present the proportion of genes as a function of the maximum expression level and tissue specificity of expression in the 53 distinct types of human normal tissues using gene expression data from the Genotype-Tissue Expression (GTEx) Project^41^. Compared to all the genes analyzed, fewer genes containing pathogenic variant(s) (the annotations are based on ClinVar database; ClinVar genes for short) show extremely high level of expression abundance and tissue-specificity as well (Fig. 3a,b, Kolmogorov-Smirnov test, p < 2 × 10^−16^), similar results were obtained in HGMD genes (genes containing DM variants annotated by HGMD). On account of the positive correlation between maximum level and tissue specificity of gene expression (Pearson’s product moment correlation coefficient, cor = 0.97 and p < 2 × 10^−16^), a straightforward explanation for this observation is that variations in genes, which are expressed with extremely high tissue specificity, are less likely disrupt the normal function of a cell; however, the possibility of damage to specific tissues which has not yet been found can neither be entirely excluded.

We further used the ranked node degree to characterize the importance of ClinVar/HGMD gene products in the overall protein-protein interaction network topology. Compare to all the genes analyzed, node degree of protein products from ClinVar/HGMD genes are generally much higher (Fig. 3c, Kolmogorov-Smirnov test, p < 2 × 10^−16^), indicating the importance for ClinVar/HGMD genes in network communication and information transfer.

We observed significant differences in gene activity between groups of genes which are classified by the number of pathogenic/DM variants detected in the exonic regions. The more sites of pathogenic variants are observed in a gene, there is more possibility that the gene is expressed in high abundance or in a high tissue-specific manner (Fig. 3d,e,g,h). Genes contain high number of pathogenic variants, their products tend to have high degree in protein-protein interaction network (Fig. 3f,i). No significant differences were observed when classifying the genes based on the density of pathogenic variants over the total exons’ length of the gene (data not shown). The data indicate that the occurrences of the pathogenic variations are non-randomly distributed in genes.

### Pathogenicity of nucleotide variations due to involvement in the regulatory process

Control of gene expression programs has an important impact on the misregulation of gene expression in disease. Many diseases and syndromes can be caused by mutations in DNA regulatory sequences^42^ as well as the regulators^43^. PolyComb group proteins modify histones and silence target genes by binding PolyComb-responsive elements^44^. As Polycomb binds to large domains that span entire gene sequence^45^, some mutations occurred in the coding region may affect the binding without disrupting the protein function. Unfortunately, most models look at the effects of DNA mutations on the shape of the protein fragment but not on the intermediate steps of transcription and translation.

**Figure 4:**
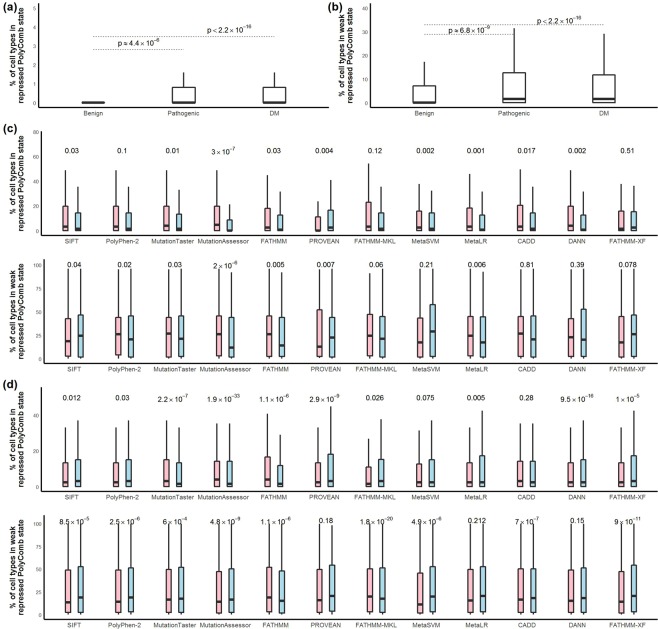
Percentage of cell types in PolyComb state for the non-synonymous sites. Sites of pathogenic variants annotated by ClinVar and DM variants annotated by HGMD are frequently found in repressed PolyComb state (**a**) as well as in weak repressed PolyComb state. (**b**) For most computational models analyzed in this study, sites of variants with false-negative predictions (red) are more frequently found in repressed or weak repressed PolyComb state compared to the sites of variants with true-positive predictions (blue) for pathogenic variants annotated by ClinVar (**c**) and DM variants annotated by HGMD. (**d**) Wilcoxon tests were used to test the significance of the differences. P-values are shown.

We, therefore, studied the preference of nucleotide sites for repressed PolyComb state taking variants pathogenicity into account. We found that more sites of pathogenic/DM variants tend to be in repressed or weak repressed PolyComb state in multiple types of cells compared to the sites of benign variants (Fig. 4a,b). One possible explanation would be, that mutation at some sites would lead to a gain-of-function due to a reprieve from the repressed state which shouldn’t happen in multiple biological conditions, consequently, the activated allele would bring a detrimental effect even though it does not destroy the protein function or not to a serious extent.

In addition, we observed that sites of false-negative variants are more frequently found in repressed or weak repressed PolyComb state compared to the sites with true-positive predictions (Fig. 4c,d). Epigenetics can affect the penetrance of genes through genomic imprinting by the paternal or maternal allele or through epigenetic regulation resulting from environmental or other personal factors. Specific DNA sequences contribute to the fidelity of epigenetic propagation and reducing spurious epigenetic inactivation events^46^. It has been reported that DNA sequence motif is required for the PolyComb proteins silencing^47^. Considering most computational tools take effects on protein function as a major criterion for classification, the data suggest that the destroyed functions due to some false-negative variants may be involved in the epigenetic process rather than in the aberrant activity of the protein product.

## Discussion

Although there have been advances in our knowledge of disease alleles, we are still far from having a complete understanding of the pathogenicity of a mutation. Our analysis adds more to our knowledge of non-synonymous SNVs’ pathogenicity, thus will benefit the field of clinical genetics.

The data suggest that existing prediction models rely too heavily on conservation scores, resulting in alarming numbers of Type I and Type II errors. We found that genomic sites show distinct levels of conservation inferred over different phylogenetic trees. Understanding this difference has important implications for the interpretation of sequencing data. Different functional elements may be constrained over different phylogenetic spans, and the depth of constraint may also vary. Nucleotides at certain genomic positions may be different along divergent branches of the vertebrate tree of life, nonetheless, show some constraint within some subset of the mammalian phylogeny, and vice versa. We suggested that conservation criteria should be taken carefully when inferring possible pathogenesis of mutations, and that a good knowledge about the evolution of the candidate genes’ function will help guide us in choosing the appropriate conservation measures to improve variant assessment.

We found that many genomic sites of false-negative variants tend to be in repressed PolyComb state in multiple types of cells. The data indicate that some pathogenic mutations may not alter the protein function, or not to a serious extent as to destroy the protein function, but lead to a defect in the epigenetic modification; the mutated allele is thus activated in multiple biological conditions resulting in disorder. Abnormal methylation, affecting multiple loci, has been identified in the genomes of patients with genetic disorders, such as a subset of Beckwith–Wiedemann syndrome and Silver–Russell syndrome patients^48,49^. It is necessary to recheck the possibility of pathogenicity of a non-synonymous SNVs which is predicted to be benign, *e.g*., the possibility of its involvement in the regulatory process that may destroy the protein function rather than the protein structure. Integrative analysis across multiple omics data along with the whole genome/exome sequencing data could help us gain a systematic perspective to identify disease-associated mutations, and it also could help generating robust and testable hypotheses.

Our work suggests a joint recommendation for the interpretation of non-synonymous variants. To implicate a variant as pathogenic requires multiple levels of evidence *i.e*. variant-level, gene-level and case-level (phenotype match). The fact that phenotype from a non-synonymous alteration of the coding gene may not constitute an adequate cause for the disease limits the accuracy of the variant-effect oriented prediction. An analysis combining variant-level features with gene-level stratification as well as combing predictions with experimental data is crucial to improve the pathogenesis interpretation of both the variant and the affected protein. Multiple classes of evidence, obtained through assays of patient-derived tissue or well-established cell or animal models of gene function, will contribute to pathogenic inferences. Such methods may not yet be applicable to every rare disease scenario, yet researchers should at the very least evaluate the variants taking advantage of public resources including genetic, informatics and experimental data.

## Materials and Methods

### Data

The annotated human (hg19) reference genome was downloaded from the UCSC Genome Browser^50^. From the UCSC Genome Browser^50^, we retrieved phyloP^32^ and phastCons^51^ conservation scores representing three different alignment types: vertebrate, primate and placental mammal for each position of variants. We collected single nucleotide variations from the 1000 Genomes Project data (phase 1)^4^, and characterized the derived allele and ancestral allele inferred from a six-way primate alignment^4^. Annotations of clinically significant variants were downloaded from ClinVar database^24^. Allele information for HGMD variants was obtained from HGMD database^25^. Epigenomic maps of histones modified by PolyComb group proteins across 127 human cells were downloaded from Roadmap Epigenomics^52^.

### Variant filtering and prioritization

Since all the prediction scores had different output scales and thus couldn’t be directly compared, we used the rank-transformed values, provided by dbNSFP^20^ to make comparisons. For each computational methods, the rankscore for a variant is the ratio of the rank of the score predicted by the method over the total number of scores in dbNSFP^20^. The FATHMM-XF^13^ predictions are fetched from the web server. Variants are predicted to be pathogenic by CADD (ranked CADD score >0.5) and DANN (ranked DANN score >0.5). The pathogenic thresholds for the rest computational methods are taken as suggested by dbNSFP^20^.

### Performance of combined tools

For the combination analysis, the criterion to categorize a variant as pathogenic or non-pathogenic is that all the algorithms tested (for each combination) agree on the prediction. We used the pathogenic thresholds for each computational tools as described above, the total number of true positives and the total number of true negatives are thus counted. The performances of all possible combinations (4095 different combinations) of the twelve tools are calculated.

### Inference of orthologous genes

Orthologues inferred from gene trees are determined based on Ensembl Genomes^30^. Orthologues are defined in Ensembl as genes for which the most common ancestor node is a speciation event. The data used in this study is based on the comparison of 137 species, including invertebrates, in Ensembl database. 1:1 orthologue means only one copy is found in each species, and 1:many orthologue means one gene in one species is orthologous to multiple genes in another species.

### Ancestral alleles of SNP sites

Ancestral alleles of SNP sites are extracted from 1000 Genomes Project data^4^. Ancestral states are inferred from the Pecan alignments. The confidence in the ancestral call is determined by comparing the call to the ancestor of the ancestral sequence as well as the ‘sister’ sequence of the query species^4^.

### Disease sensitivity genes

Disease-sensitive genes are defined as genes containing high proportion (>30%) of pathogenic variations annotated by ClinVar or HGMD. Disease-tolerant genes are defined as genes where no pathogenic variations was annotated neither by ClinVar database nor by HGMD database so far, while more than fifty polymorphic sites were detected by the 1000 Genomes Project.

### Abundance and tissue-specificity of gene expression

We downloaded normalized gene expression data for 53 distinct types of human normal tissues from the Genotype-Tissue Expression (GTEx) Project^41^. To avoid variability in gene expression patterns between different experimental or biological conditions, the maximum abundance among the tissues is used to measure gene expression level. We calculated the expression specificity of a gene according to the information content^53^, ${\mathrm{log}}_{2}n+\sum _{i=1}^{n}{p}_{i}{\mathrm{log}}_{x}{p}_{i}$, where *n* is the number of tissues, and *p*_*i*_ is the percentage of expression abundance in tissue *i*.

### Functional enrichment of genes

Gene Ontology (GO) enrichment analysis was performed using DAVID (http://david.abcc.ncifcrf.gov)^54^ using Ensembl Gene IDs and the entire human genome as a background model. Gene set enrichment analysis (GSEA) was performed using preexisting human gene set annotations from the Broad Institute^31^. P-values were adjusted by FDR.

### Maximum and coefficient of variation of prediction scores for the non-degenerate sites

We chose the maximum of the prediction scores assigned to the four different nucleotides, *i.e*. A/T/G/C, to evaluate prediction performance on the non-degenerate sites. The coefficient of variation (CV) of the prediction scores assigned to the four different nucleotides at a non-degenerate site is calculated as $CV=\sigma /\mu$^55^, ${\rm{\sigma }}=\sqrt{\frac{{\sum }_{i}{({{\rm{S}}}_{i}-{\rm{\mu }})}^{2}}{3}},\,\mu =\frac{{\sum }_{i}{{\rm{S}}}_{i}}{4}$, where *S*_*i*_ is the prediction score of nucleotide *i*, and $i\in ({\rm{A}},{\rm{T}},{\rm{G}},{\rm{C}})$.

All statistical analysis were performed using the computing environment R^56^.

## Supplementary information

Supplementary file
