# Supplementary material for: New insights into the pathogenicity of non-synonymous variants through multi-level analysis
Source: Sci Rep. 2019 Feb 7;9:1667. doi: 10.1038/s41598-018-38189-9 (PMC6367327; doi:10.1038/s41598-018-38189-9)
Supplement: Supplementary file 1 — Supplementary file [file 41598_2018_38189_MOESM1_ESM.pdf]

# Supplemental Materials for

# New insights into the pathogenicity of non-synonymous variants through multi-level analysis

Hong Sun[1] and Guangjun Yu[1*]
[1] Children's Hospital of Shanghai, Shanghai Jiao Tong University, Shanghai, China

***** Guangjun Yu, E-mail: gjyu@shchildren.com.cn

This file includes Supplemental Tables S1 and Supplemental Figures S1 to S15.

## Supplementary Tables

**Supplementary Table S1**. Performance of the computational tools.

| Tools | Accuracy | Sensitivity | Specificity |
|---|---|---|---|
| PROVEAN | 0.557 | 0.923 | 0.283 |
| MutationTaster | 0.623 | 0.889 | 0.424 |
| FATHMM-MKL | 0.672 | 0.941 | 0.471 |
| PolyPhen-2 | 0.739 | 0.863 | 0.645 |
| MutationAssessor | 0.743 | 0.659 | 0.806 |
| SIFT | 0.744 | 0.887 | 0.636 |
| DANN | 0.754 | 0.808 | 0.714 |
| CADD | 0.758 | 0.866 | 0.678 |
| FATHMM | 0.767 | 0.561 | 0.921 |
| MetaLR | 0.784 | 0.566 | 0.946 |
| MetaSVM | 0.816 | 0.665 | 0.929 |
| FATHMM-XF | 0.797 | 0.793 | 0.799 |

## Supplementary Figures

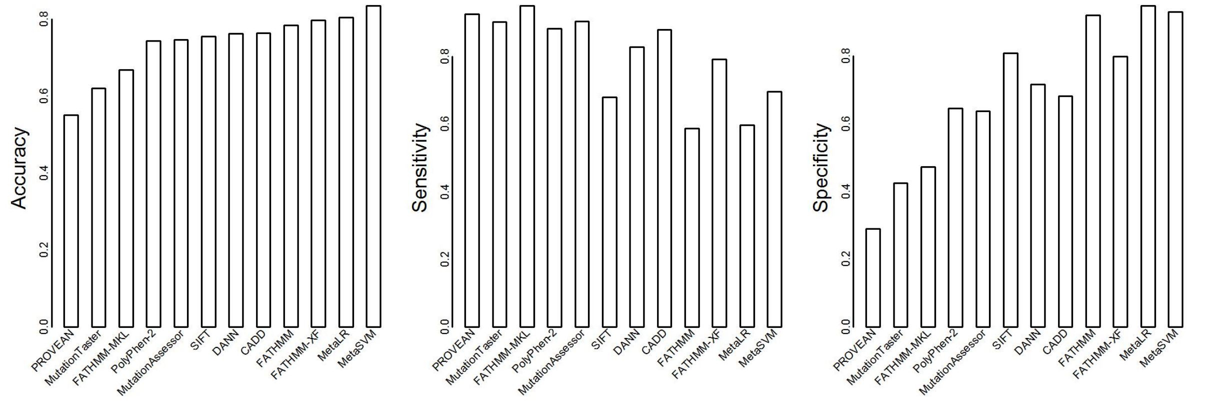


**Supplementary Figure S1**. Performance of the computational tools. The test data is based on ClinVar. A total of 10,606 sites were left for the analysis after excluding sites with conflicting annotations.

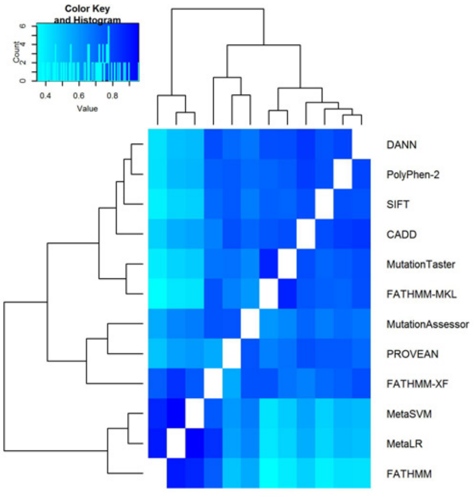


**Supplementary Figure S2**. Proportion of variants that have consensus predictions in pairwise testing.

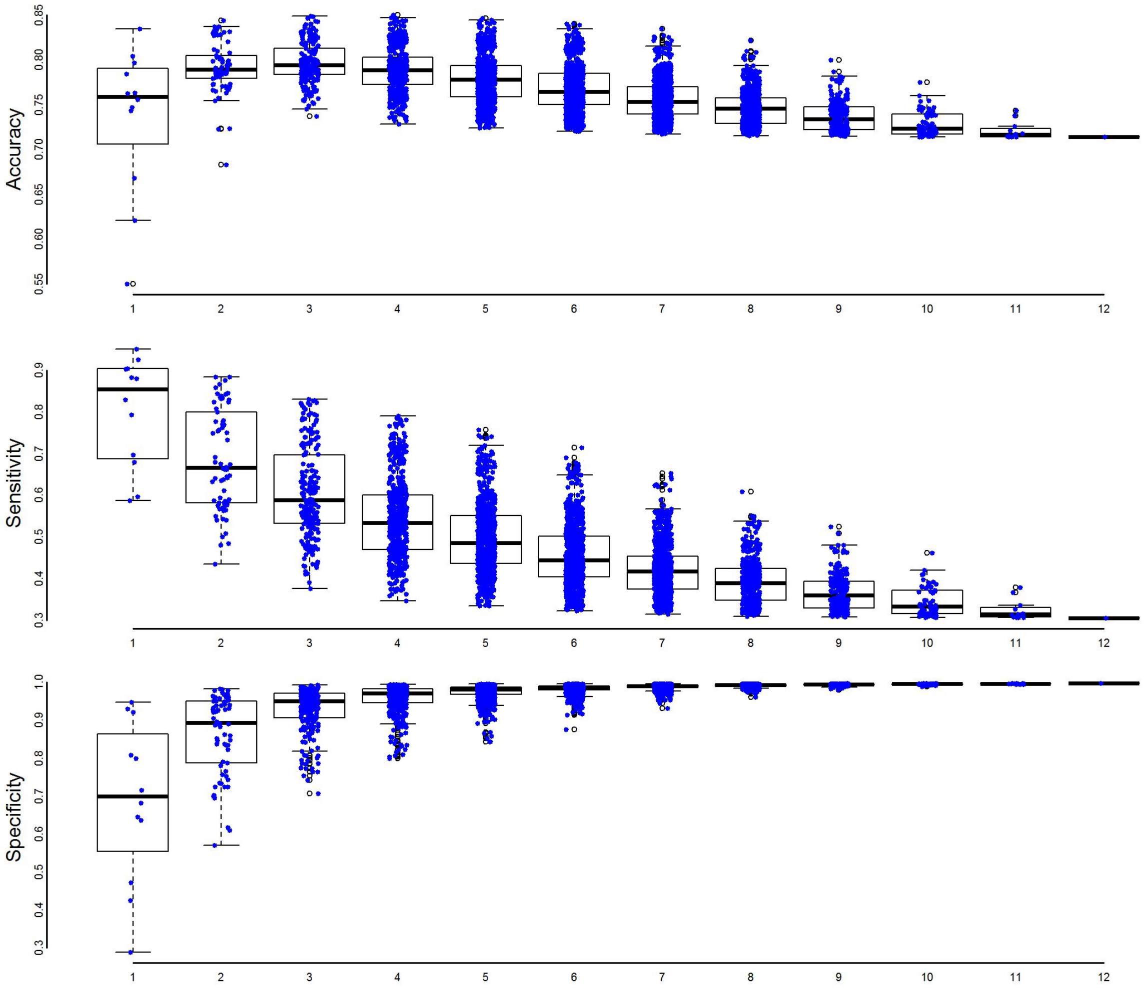


**Supplementary Figure S3**: Performance of the combined predictions corresponding to the number of algorithms combined. The criteria to categorize a variant as pathogenic or non-pathogenic is that all the combined algorithms agree on the prediction.

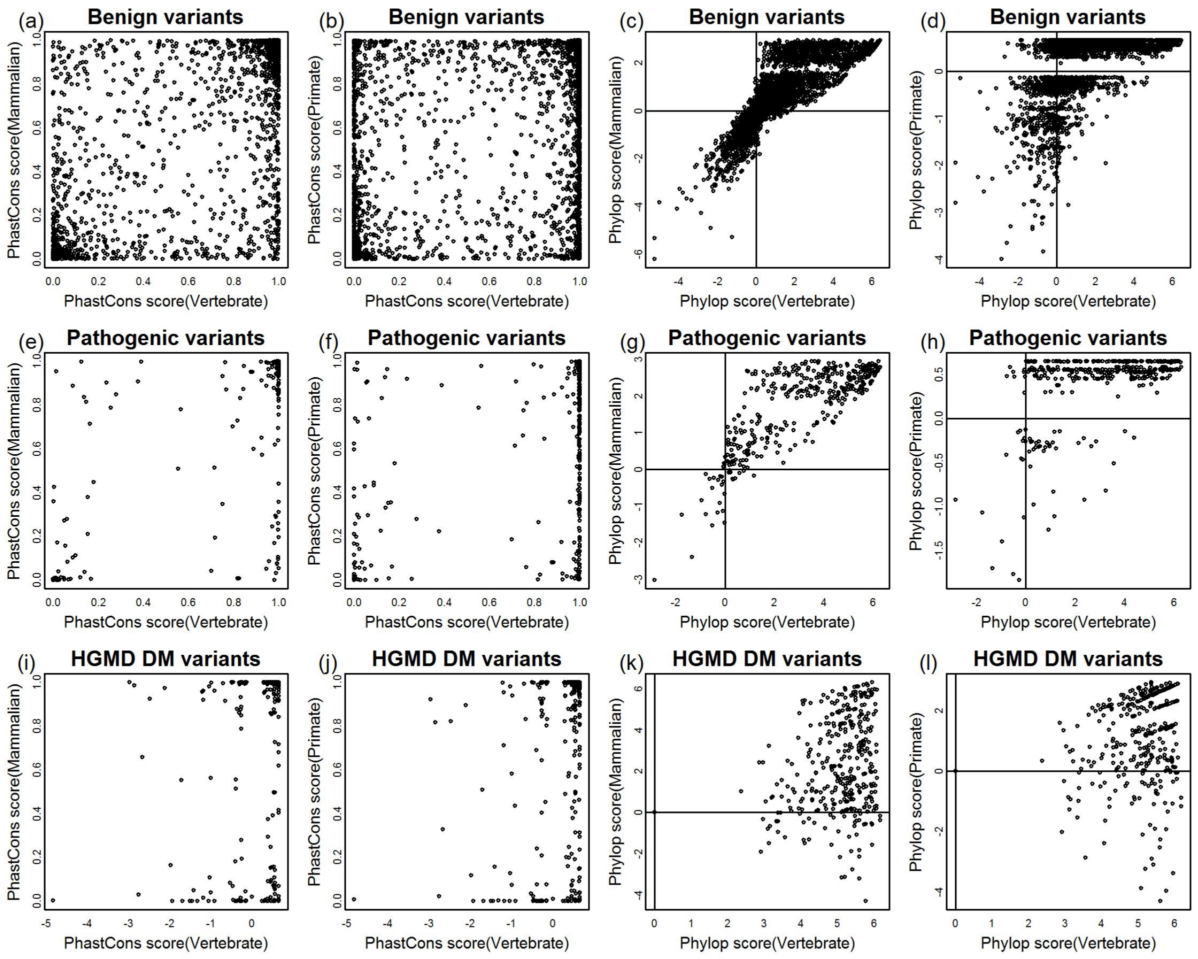


**Supplementary Figure S4**. Evolutionary conservation measured by PhastCons scores and Phylop scores across vertebrates, across mammalians or across primates for positions of benign variants (**a, b, c, d**), for positions of pathogenic variants (**e, f, g, h**) annotated by ClinVar and for positions of DM variants (**i, j, k, l**) annotated by HGMD.

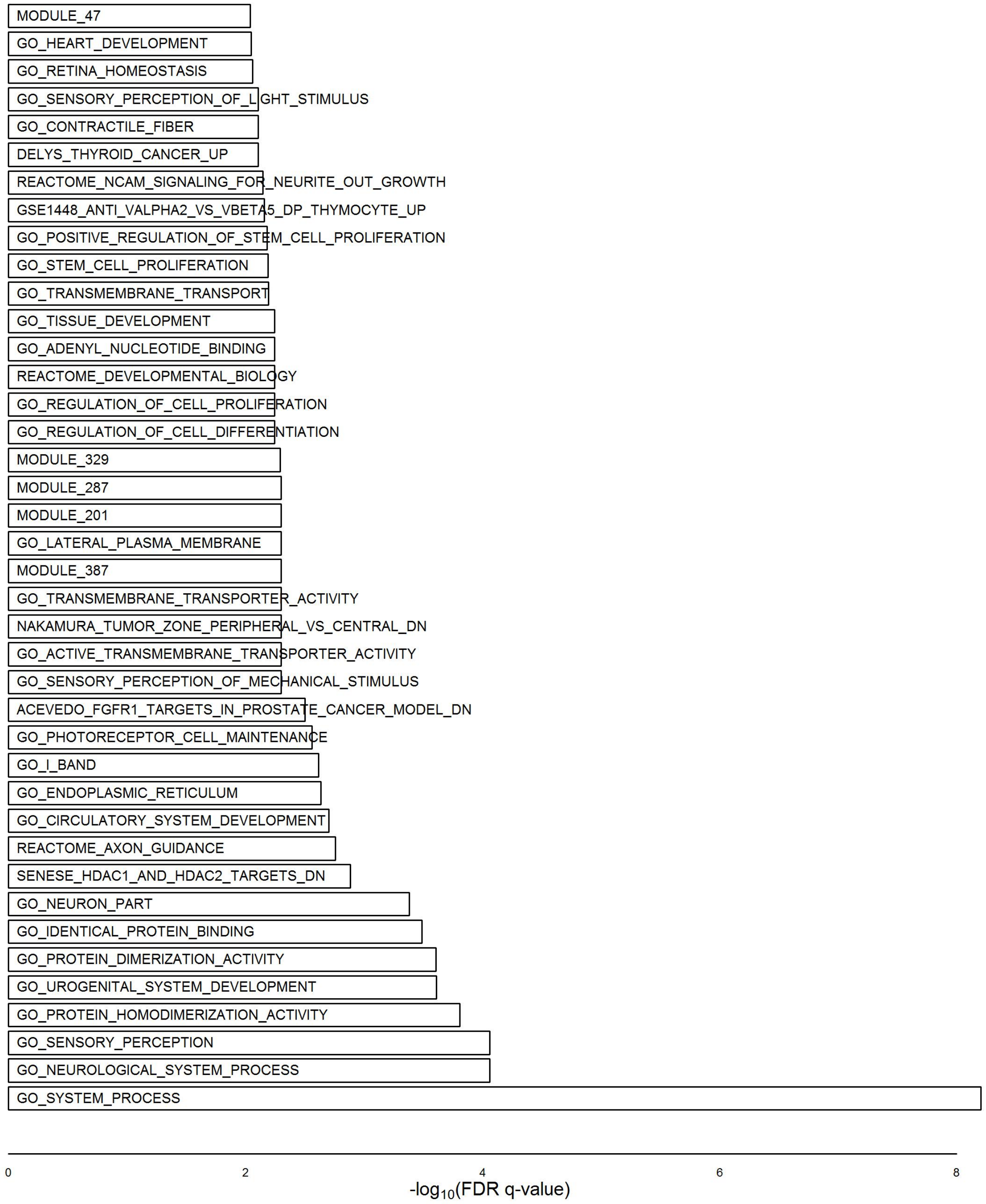


**Supplementary Figure S5**. Summary of GSEA analysis performed on the thirty-three genes where ancestral alleles were identified as pathogenic variants by ClinVar or DM variants by HGMD.

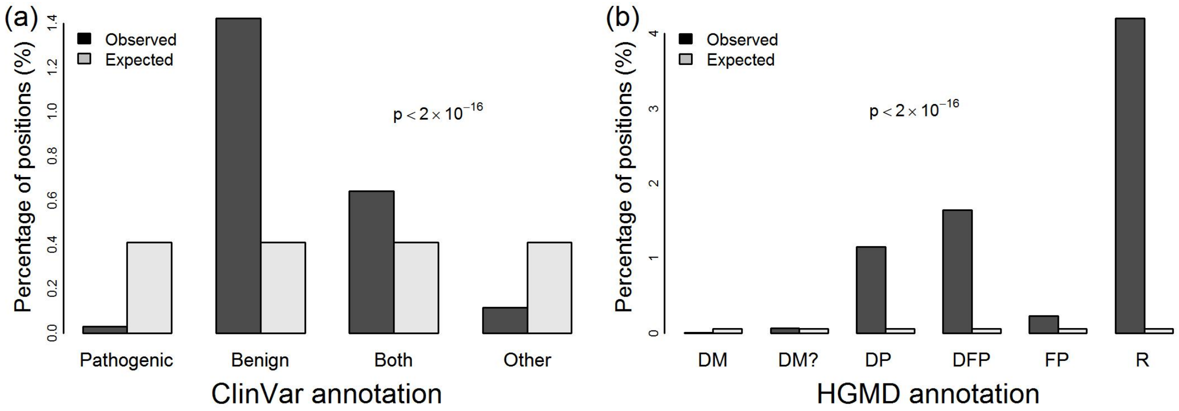


**Supplementary Figure S6**. Percentage of the mutations that change back to the ancestral state corresponding to all the variant positions of the four classes of ClinVar annotations (**a**) and to all the variant positions of the six classes of HGMD annotations (**b**). Abbreviations for ClinVar annotation items: pathogenic sites if pathogenic evidence was presented to the change(s) at the site but no evidence of benign effect from an authoritative source, and for the benign sites vice versa; a site is called as ‘both’ (pathogenic or benign) if both pathogenic and benign variants were found, and a site is called as ‘other’ if neither pathogenic nor benign variants were found. Abbreviations for HGMD annotation items: DM: Disease causing mutation; DM?: Disease causing mutation?; DP: Disease-associated polymorphism; DFP: Disease-associated polymorphism with supporting functional evidence; FP: In vitro/laboratory or in vivo functional polymorphism; R: Retired entry (R). We used the chi-square test of independence to test for equality of proportions, p-value are shown.

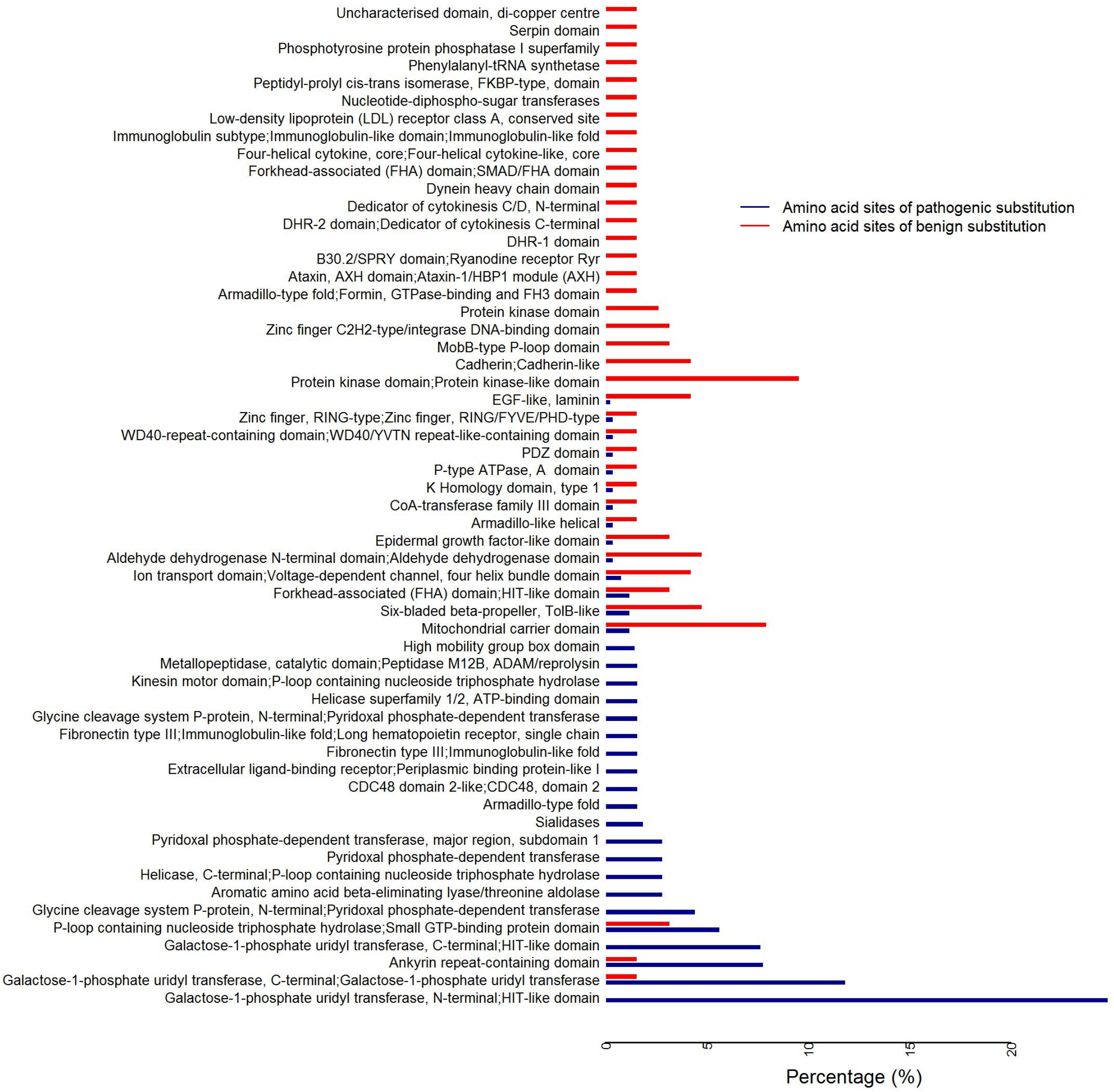


**Supplementary Figure S7**. Percentage of the amino acid substitutions originated from mutations at non-degenerate sites corresponding to protein domains.

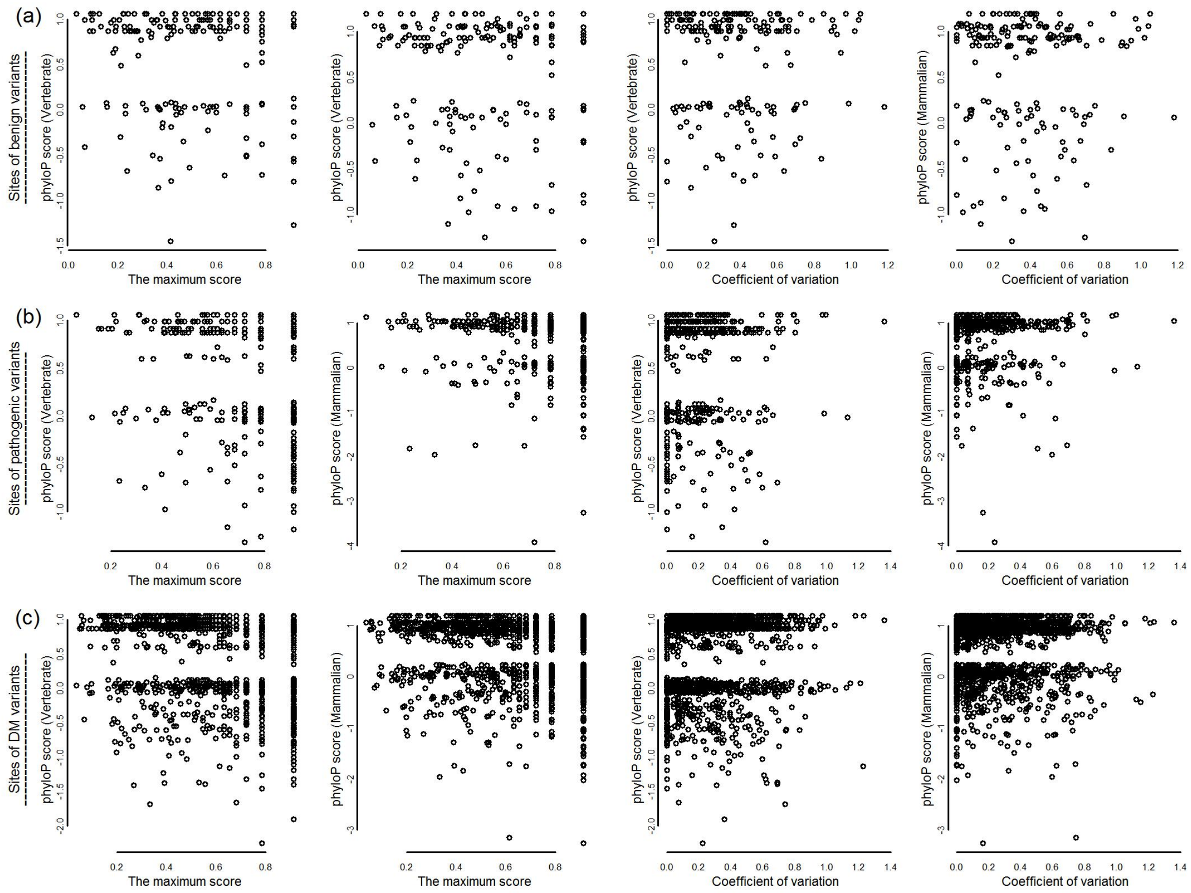


**Supplementary Figure S8**. Relationship between predication scores and conservation scores for the non-degenerate sites of pathogenic variants (**a**), for the non-degenerate sites of benign variants (**b**) based on the ClinVar annotation, and for the non-degenerate sites of DM variants based on the HGMD annotation (**c**). The maximum prediction score of the four different nucleotides and the coefficient of variation of the four prediction scores are derived from predictions of the SIFT program as an example.

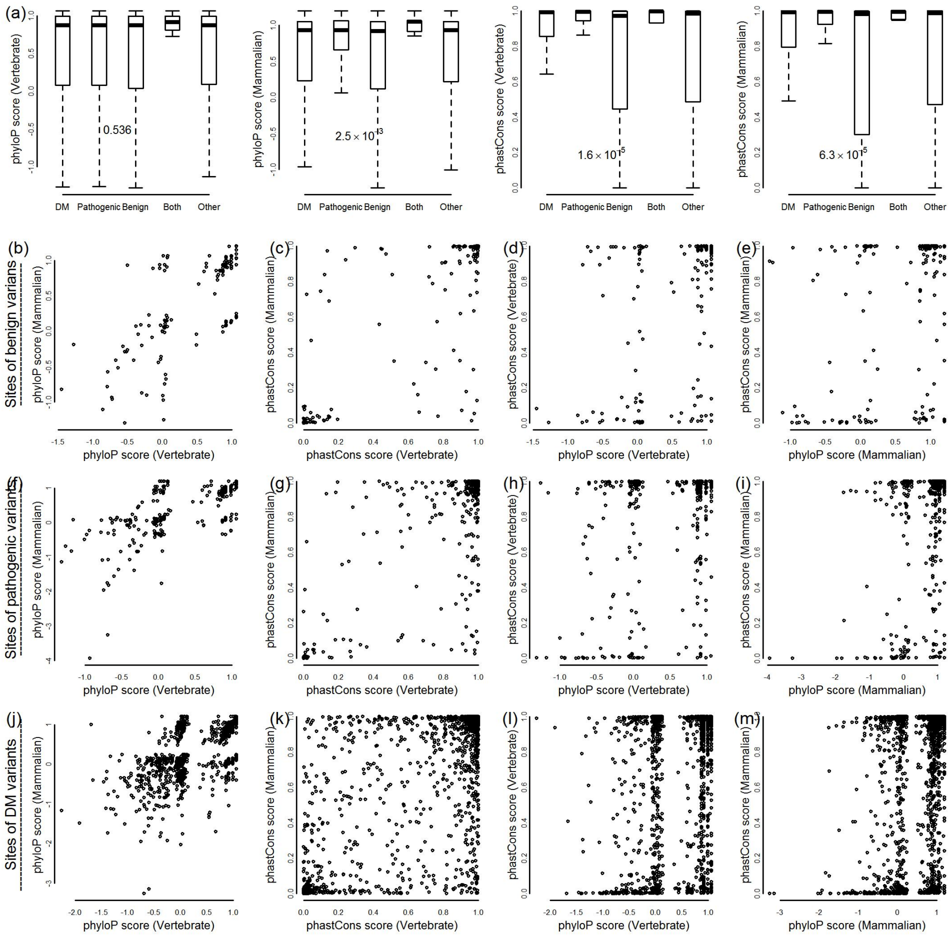


**Supplementary Figure S9**. Conservation analysis of the non-degenerate sites. Conservation scores based on four conservation measurements, *i.e.* phastCons scores and phyloP scores derived from vertebrates or placental mammals model (**a**). The p-values of differences between sites of pathogenic variants and sites of benign variants are shown. Scatterplot of phyloP scores and phastCons scores derived from vertebrates model against placental mammals model for sites of pathogenic variants (**b, c**), benign variants (**f, g**), and for sites of DM variants (**j, k**). Scatterplot of phyloP scores against phastCons scores for sites of pathogenic variants (**d, e**), benign variants (**h, i**), and for sites of DM variants (**l, m**).

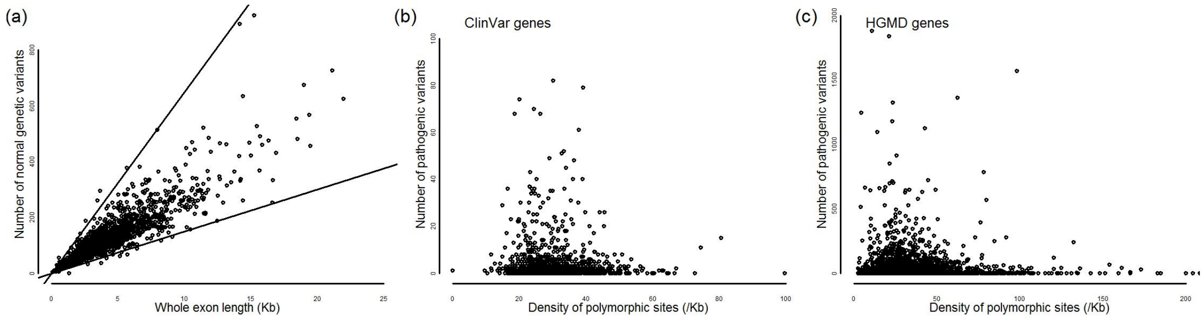


**Supplementary Figure S10**: An overview of genetic burden on human genes. **(a)** Occurrence of genetic variations in genes over the whole exons' length. **(b, c)**, Number of sites of pathogenic variants corresponding to the density of polymorphic sites. The annotation of pathogenicity is based on ClinVar database and HGMD database and the number of observed polymorphic sites in genes is calculated based on the 1000 Genomes Project data (phase 3).

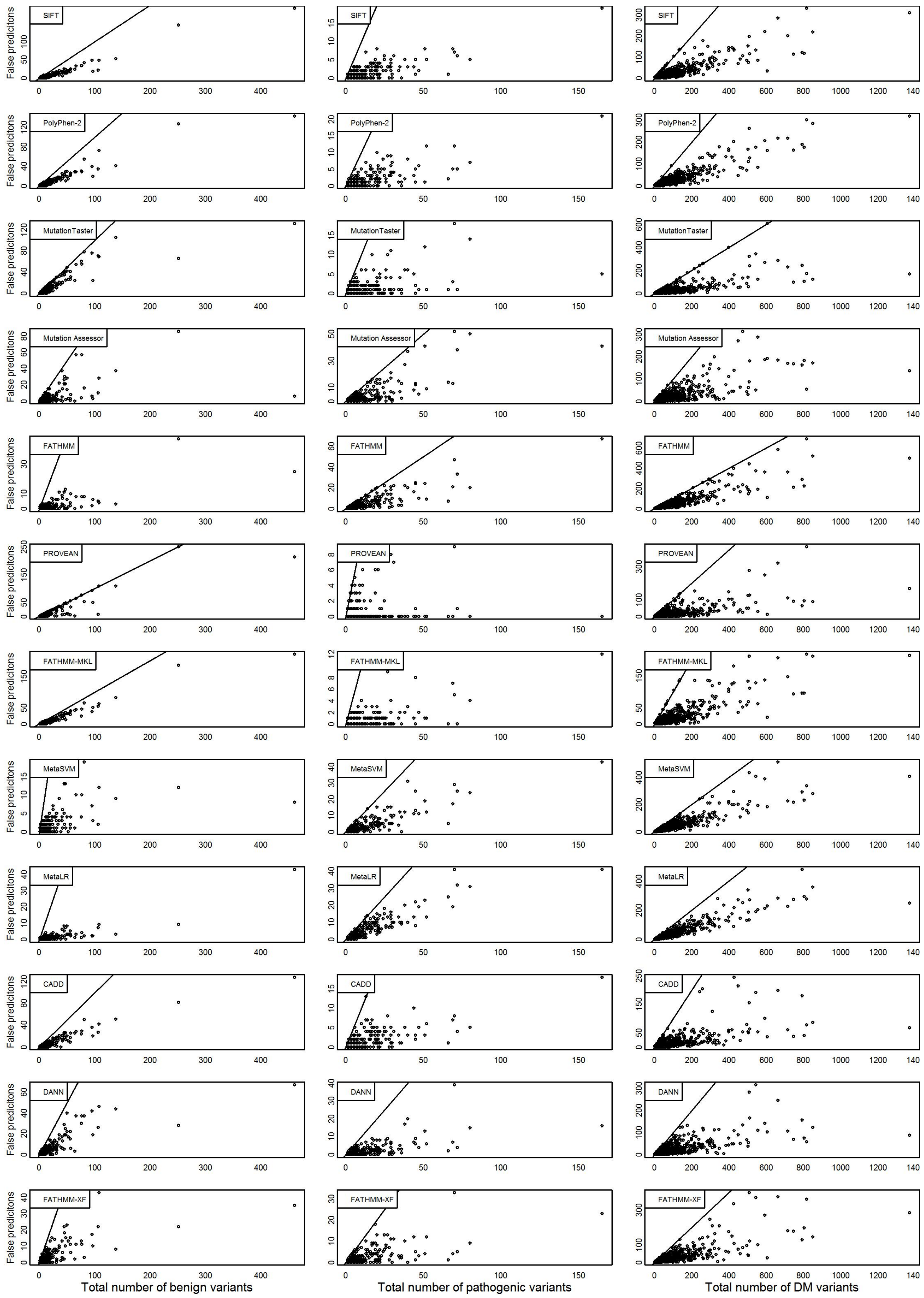


**Supplementary Figure S11**: Number of false predictions corresponding to the total number of benign or pathogenic variants in genes annotated by ClinVar database and corresponding to the total number of DM variants in genes annotated by HGMD database.

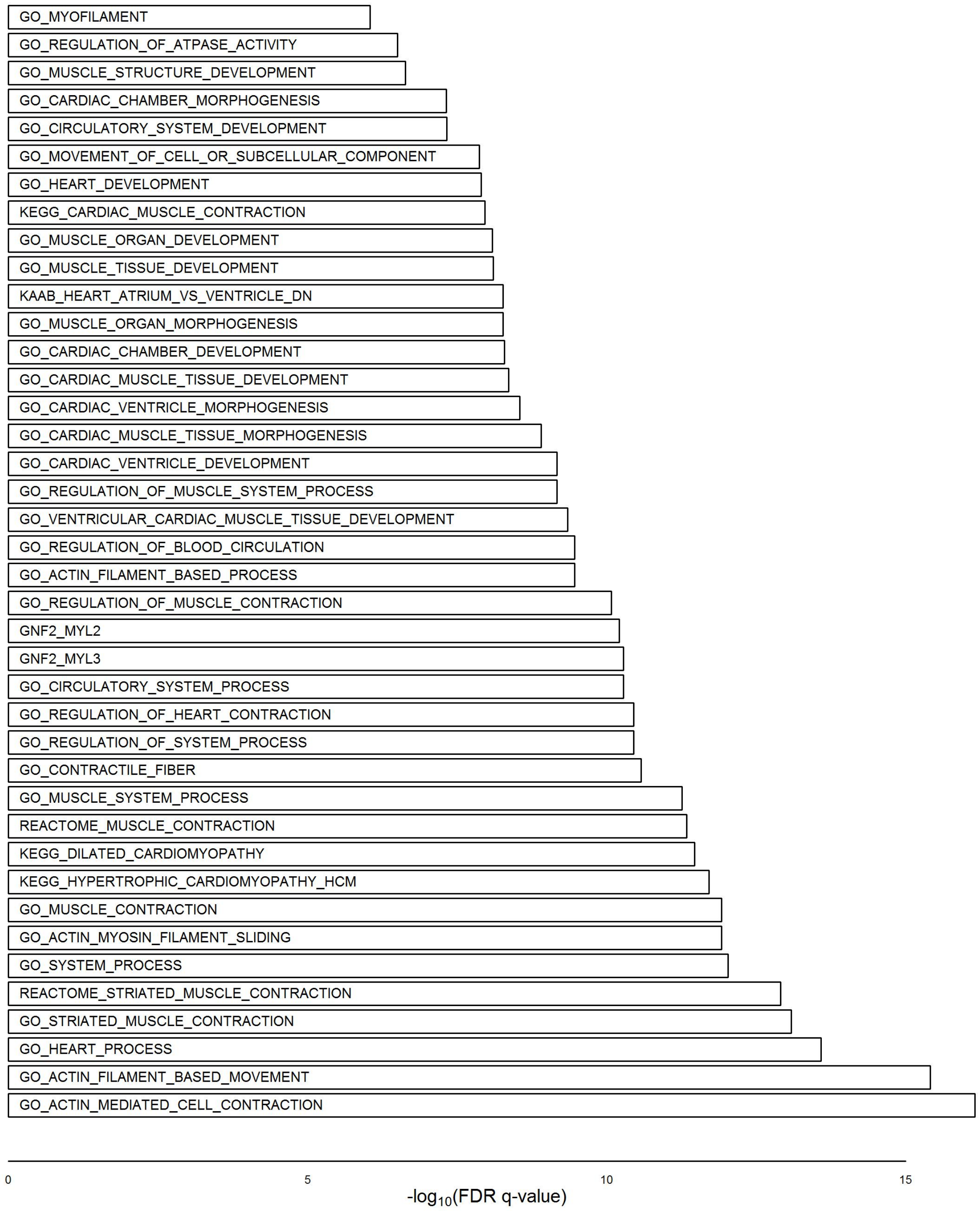


**Supplementary Figure S12**. Summary of GSEA analysis performed on disease-sensitive genes. Disease-sensitive genes are defined as genes containing high proportion (> 30%) of pathogenic variations annotated by ClinVar database or by HGMD database.

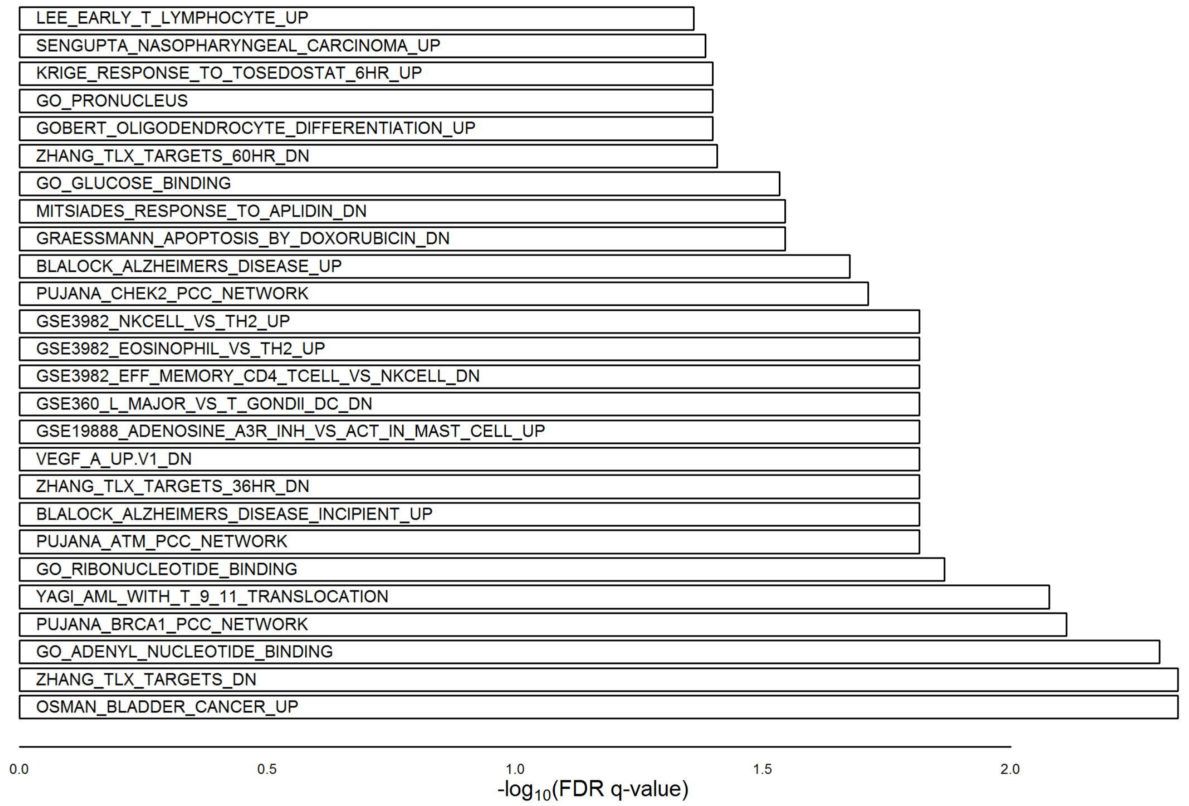


**Supplementary Figure S13**. Summary of GSEA analysis performed on disease-tolerant genes. Disease-tolerant genes are defined as genes where no pathogenic variations were annotated neither by ClinVar database nor by HGMD database so far, while more than fifty polymorphic sites were detected by the 1000 Genomes Project.

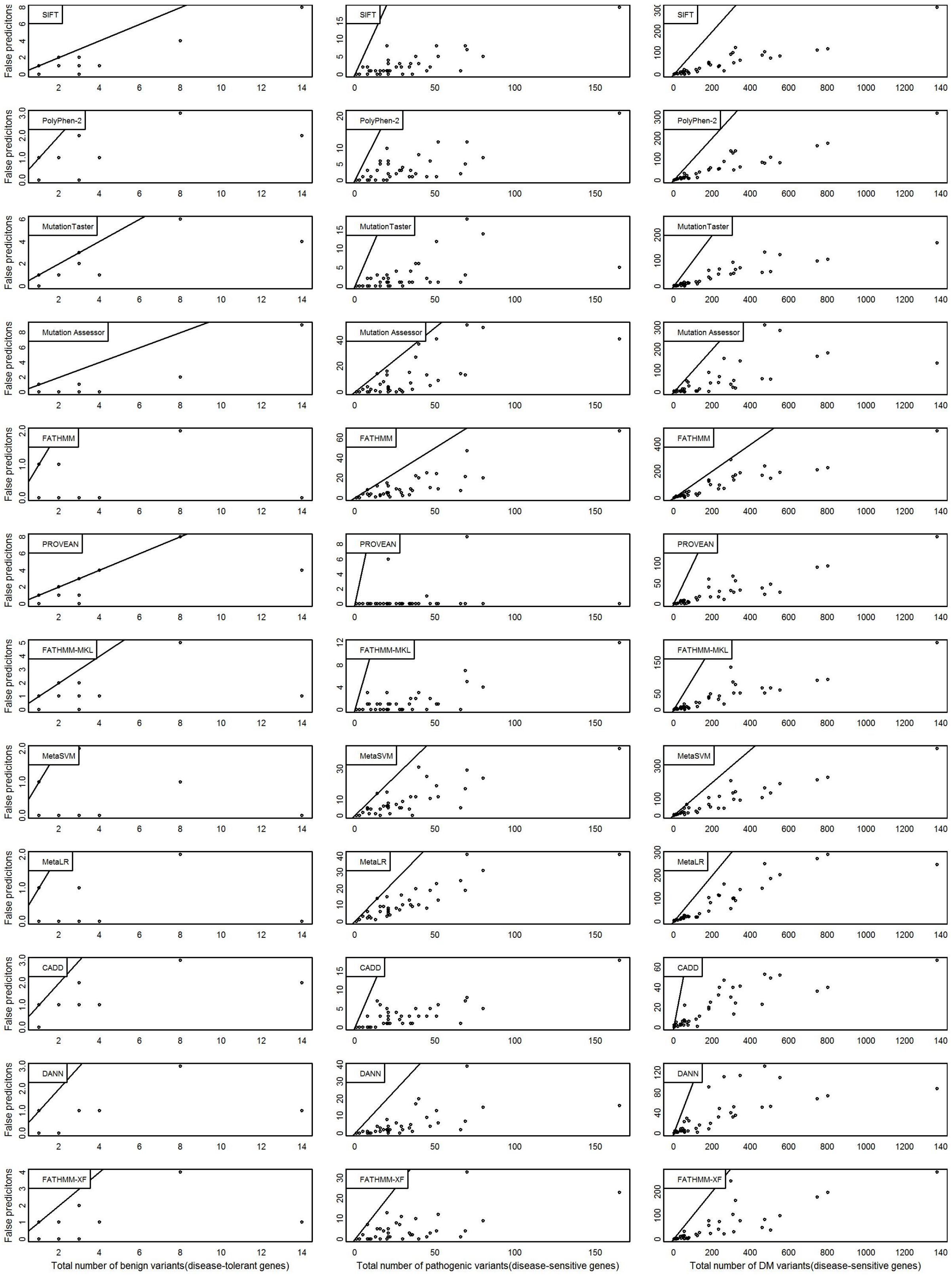


**Supplementary Figure S14**: Number of falsely predicated variants corresponding to the total number of pathogenic variants, benign variants, and DM variants in genes with high or low disease susceptibility. In this analysis, disease-sensitive genes are defined as genes containing high proportion of sites of pathogenic variants (> 30%), and disease-tolerant genes are defined as genes where no pathogenic variants were observed so far meanwhile more than fifty of genetic variants were found.

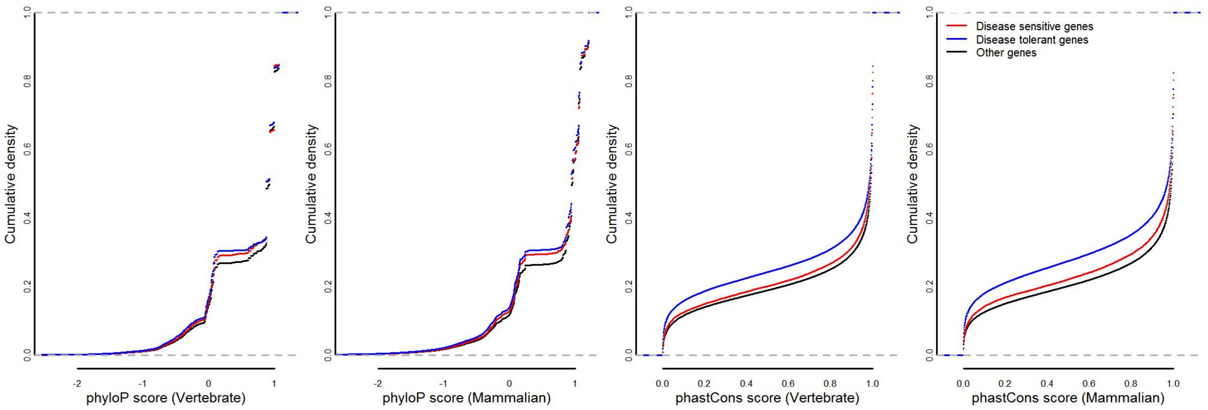


**Supplementary Figure S15**. Cumulative frequency of nucleotides located in disease-sensitive genes, disease-tolerant genes and other genes (all the other genes out of disease-sensitive genes and disease-tolerant genes) corresponding to conservation scores. Kolmogorov-Smirnov tests were used to test the significance of the differences between gene groups, and significant differences ($p < 2\times10^{-16}$) were observed across all the pairwise comparisons.
